# Stable Long-Term Culture of Human Distal Airway Stem Cells for Transplantation

**DOI:** 10.1155/2021/9974635

**Published:** 2021-09-16

**Authors:** Yueqing Zhou, Yujia Wang, Dandan Li, Ting Zhang, Yu Ma, Wei Zuo

**Affiliations:** ^1^East Hospital, Tongji University School of Medicine, Shanghai 200120, China; ^2^Regend Therapeutics, Shanghai 200127, China; ^3^Ningxia Medical University, Yinchuan 750004, China; ^4^The First Affiliated Hospital, Guangzhou Medical University, Guangzhou 510120, China

## Abstract

There is a population of p63^+^/Krt5^+^ distal airway stem cells (DASCs) quiescently located in the airway basal epithelium of mammals, responding to injury and airway epithelial regeneration. They hold the ability to differentiate into multiple pulmonary cell types and can repopulate the epithelium after damage. The current study aims at gaining further insights into the behavior and characteristics of the DASCs isolated from the patient lung and exploring their clinical translational potential. Human DASCs were brushed off through the bronchoscopic procedure and expanded under the pharmaceutical-grade condition. Their phenotype stability in long-term cell culture was analyzed, followed by safety evaluation and tumorigenic analysis using multiple animal models including rodents and nonhuman primate. The chimerism of the human-mouse lung model indicated that DASC pedigrees could give rise to multiple epithelial types, including type I alveolar cells as well as bronchiolar secretory cells, to regenerate the distal lung. Taken together, the results suggested that DASC transplantation could be a promising therapeutic approach for unmet needs in respiratory medicine including the COVID-19-related diseases.

## 1. Introduction

The lung is a complex organ that takes responsibility for gas exchange, including filtering and delivering inhaled and exhaled air [1]. Lung diseases constitute a serious threat to human public health worldwide, with high morbidity and mortality [2–6]. Despite that mitigating therapies contribute to control deterioration, it remains limited to repair and recover the pulmonary function of lung disease particularly such as bronchiectasis, idiopathic pulmonary fibrosis (IPF) [7], and chronic obstructive pulmonary disease (COPD) [8, 9], which involves the progressive and inexorable destruction of oxygen exchange surfaces and airways.

Multiple stem/progenitor populations [10–15] in the lung with the capability to reconstruct lung epithelium have been identified in the last decade, which can be regarded as a potential candidate for therapeutic strategies targeted to damages of airway and alveolar tissues. Previously, we showed that a rare population of distal airway stem cells (DASCs) identified coexpressing p63^+^/Krt5^+^, quiescently located at the airway basal epithelium of mammals, responding to injury and airway epithelial regeneration [16–21]. They hold the ability to differentiate into multiple pulmonary cell types and repopulate the epithelium after damage. DASCs undergo a proliferative expansion and migration in response to influenza-induced or bleomycin-induced lung damage and assemble into nascent alveoli at sites of interstitial lung inflammation [17, 20, 21]. Human DASCs can be cloned *in vitro* and xenotransplanted into the murine lung, giving rise to “human-mouse chimeric lung” [16].

In the current study, to gain further insights into the behavior and character of the DASCs isolated from the patient lung, we first analyzed their stability in cell culture under the pharmaceutic-grade condition as a cell therapeutical candidate, followed with valuation of their safety and efficacy using multiple animal models, including a rodent model and nonhuman primate model. Taken together, we provide an all-around evaluation of human DASCs for further clinical trials utilizing autologous lung stem/progenitor cells as a therapeutic intervention in multiple respiratory diseases.

## 2. Method

### 2.1. Animals

Cynomolgus macaque (*M. fascicularis*), aged 60 months and originating from Guangdong Qianyan Biological Science and Technology Co. Ltd., was used in this study. Macaque was housed in JOINN Laboratories infrastructure facilities (Suzhou) in compliance with Animal Welfare Act and Regulations (Public Law 99-198) promulgated by USDA. The protocols were approved by the institutional ethical committee under statement number ACU18-959. NOD-SCID mice (6–8 weeks old) purchased from Shanghai SLAC Laboratory Animal Co. Ltd. (China), maintained in SPF animal facilities, were used for human DASC transplantation.

### 2.2. Isolation and Culture of Human Distal Airway Stem Cells

Patients diagnosed with or without chronic lung diseases (COPD, bronchiectasis, and ILD), through the ATS/ERS guideline, were recruited. All individuals went through thorough medical examination before sampling. Human distal airway stem cells located were brushed off through bronchoscopic procedure performed by board-certified respiratory physicians using a flexible fiberoptic bronchoscope (Olympus, Japan) [16]. Briefly, after oropharyngeal and laryngeal anesthesia, the bronchoscope was advanced through the vocal cords with 2 mL 2% lidocaine solution instilled into the trachea and both main bronchi. A disposable 2 mm brush was advanced through the working channel of the bronchoscope and used to collect lung epithelial samples by gently brushing back and forth 1–2 times in the 4~6th-order bronchi. Samples were digested, passed through a 70 *μ*m cell strainer, and washed with cold DMEM medium supplemented with clinically approved antibiotics. The cell suspension was plated onto irradiated 3T3 fibroblast feeder cells from ATCC CCL­92 and cultured in pharmaceutical-grade culture medium, including DMEM/F12, 10% FBS (HyClone, Australia), Pen/Strep, amphotericin, and growth factor cocktail as previously described [16] with 7.5% CO_2_ for DASC-selective growth and expansion. DASCs were grown in primary cultures with antibiotics and continuously propagated in the following feeder-free cultures in the absence of antibiotics. Then, cells were harvested, washed, and suspended in clinically approved 0.9% *w*/*v* of NaCl. The harvested cells were directly used for preclinical experiments.

### 2.3. In Vitro Monolayer Differentiation

The monolayer differentiation system *in vitro* was described previously [20]. Cells from two donors were first cultured in culture medium for 1 day and then transferred to serum-free DMEM/F12 medium supplemented with FGF10 (50 ng/mL, PeproTech, USA), transferrin (5 *μ*g/mL, PeproTech, USA), HGF (20 ng/mL, PeproTech, USA), and 5% BSA for 5 days to induce distal lung differentiation [22].

### 2.4. Immunofluorescence

Fresh tissue was fixed in 4% paraformaldehyde (PFA) overnight at 4°C and then settled by 30% sucrose before embedding into the Tissue-Tek O.C.T. compound (Sakura, Japan). All the samples were sliced into 5–7 *μ*m thickness using a microtome (Leica Microsystems, Germany).

Immunofluorescence staining was conducted by the standard protocol described previously [16]. Cells attached on a plate or tissue sections were fixed by 3.7% formaldehyde and then incubated with 0.2% Triton X-100 to improve the cell permeability for 10 min. Tissue slices were subjected to antigen retrieval in citrate buffer (pH 6.0, Sigma, USA) in the microwave oven for 20 min before staining. Primary antibodies were incubated overnight at 4°C, following 10% donkey serum blocking for 2 h at RT. Antibodies used in the current study were anti-KRT5 (1 : 200, EP1601Y, Thermo Fisher Scientific, USA), anti-P63 (1 : 500, 4A4, Abcam, USA), anti-Ki67 (1 : 500, B126.1, Abcam, USA), anti-SCGB1A1 (1 : 200, T-18, Santa Cruz Biotechnology, USA), anti-human specific Lamin A+C (1 : 200, ab108595, Abcam, USA), anti-AQP5 (1 : 500, EPR3747, Abcam, USA), anti-HOPX (1 : 200, ab230544, Abcam, USA), anti-PDPN (1 : 200, 18H5, Santa Cruz Biotechnology, USA), anti-GFP (1 : 200, ab290, Abcam, USA), and anti-GFP (1 : 500, ab6673, Abcam, USA). Alexa Fluor-conjugated 488/594 (1 : 500, Life Technologies, USA) antibodies were used as secondary antibodies.

### 2.5. Tumorigenic Assay

For anchorage-independent growth assay, 0.75 × 10^4^ cells from 2 donors were seeded in 1 mL of a 0.375% upper agar (Sigma) layer on a 0.5% under agar layer in the DMEM supplemented with 10% FBS. Cultures were usually maintained for 14 days, and then, gels were stained by crystal violet-methanol solution (SolarBio) [19, 23].

A total of 15 male NOD-SCID mice (6–8 weeks old) were used for *in vivo* tumorigenic assay. Mice were equally divided into 3 groups and received a subcutaneous injection of either 10^7^ DASCs from 2 donors, 10^7^ human embryonic lung fibroblast cell line MRC-5, or 10^6^ HeLa cells. Tumor sizes were measured with a caliper at the injection site on indicated time points. The tumor volume was calculated using the formula volume = 0.5 × length (mm) × (width [mm])^2^. Mice that had tumors with the longest diameter of 20 mm or with a sign of physiological decondition were euthanized, necropsied, and subjected for gross observation and histopathological examination. The last measurement was carried forward for the mice euthanized on day 112.

### 2.6. DASC Transplantation Experiment

NOD-SCID mice (6–8 weeks old) purchased from Shanghai SLAC Laboratory Animal Co. Ltd. (China), maintained in SPF animal facilities, were used for human DASC transplantation. The mouse lung was injured by intratracheally instilling with bleomycin (3 U/kg body weight, SelleckChem, USA) 8 days before cell transplantation. Then, mice were anesthetized by intraperitoneal injection of 1.25% avertin and rested on a stand gesture. One million DASCs suspended in 50 *μ*L of PBS were intratracheally instilled into the injured lung. On indicated day posttransplantation, mice were euthanatized and the lung samples were harvested for immunofluorescence analysis. Bright-field and direct fluorescence images of the transplanted lung were acquired under the fluorescence stereomicroscope (MVX10, Olympus, Japan).

In Vivo Safety Assay of hDASCs

To test the potential acute toxicity of cells *in vivo*, a preclinical short-term safety assay was conducted by single intratracheal administration of hDASCs from 2 donors, containing at least 35-fold higher than the intentional clinical dose in male mice (8–9 weeks old). In detail, 20 mice were equally divided into the three hDASC groups, which received cell transplantation at a dose level of 6 × 10^6^ cells/kg (low dose), 3 × 10^7^ cells/kg (medium dose), and 1.5 × 10^8^ cells/kg (high dose), and a control group received normal saline. The morbidity, mortality, abnormal behavior, and toxic reactions, if any, were observed for 14 days after the transplantation. After the 14-day observation period, all mice were euthanized, necropsied, and subjected for gross observation and histopathological examination.

For the preclinical long-term safety assay, a total of 80 male mice were equally divided into two groups: a cell treatment group that received hDASCs from 3 donors or a control group that received normal saline. Mice in the cell treatment group received two deliveries of hDASCs at a dose level of 6 × 10^7^ cells/kg/delivery on day 0 and day 28. Changes in fur, skin, limbs, mouth, nose, and eyes; abnormal behavior in physical, physiological, or neurological activities; and changes in reactivity to handling or sensory stimuli, if any, were recorded daily. Bodyweight and food/water intake were recorded weekly. Twenty mice from each group were necropsied and subjected to multiple examinations by the end of the administration period (day 30), and the left mice were necropsied and subjected for examination by the end of the observation period (day 57). A whole set of the examination includes hematologic profiling, lymphocyte subset counting, blood coagulation test, serum biochemistry analysis, and quantification of serum immunoglobulin, complement, and inflammatory cytokines. The gross necropsy and histopathological examination of organs, which included the brain, heart, lungs, trachea, kidneys, liver, spleen, testis, and bone marrow, were carried out.

### 2.7. Nonhuman Primate Model for Transplantation

One macaque was pretreated by the electron linear accelerator radiation (3.12 Gy and 1.55 Gy/min) [24] 7 days prior to cell transplantation, using ketamine (10 mg/mL) and pentobarbital sodium (15 mg/mL) as anesthesia. Cell suspension (10^7^ cells/mL and 5 mL/kg) was infused into lobes through bronchoscopy performed by respiratory physicians. Seven days posttransplantation, Macaque was euthanatized and all organ samples were harvested for immunofluorescence analysis. During the experiment, food intake and general clinical observation were daily monitored and detailed clinical observation, such as limbs, breathing, and skin, was weekly examined. Bright-field and direct fluorescence images of the transplanted lung were acquired under the fluorescence stereomicroscope (MVX10, Olympus, Japan).

### 2.8. Statistics

Continuous data are presented as mean ± standard deviation and categorical data as an absolute number and percentage of patients in each category. Preclinical data were first assessed by Levene's test for normality. Comparison between groups was assessed through unpaired *t*-test or Mann–Whitney *U*-test according to normality. Changes in tumor volume in the tumorigenic assay were analyzed by repeated measurement ANOVA, with a *p* value showing the significance of between-subjects effects.

## 3. Results

### 3.1. Bronchoscopic Isolation and Stable Expansion of hDASC from Patient Lung

**Figure 1:**
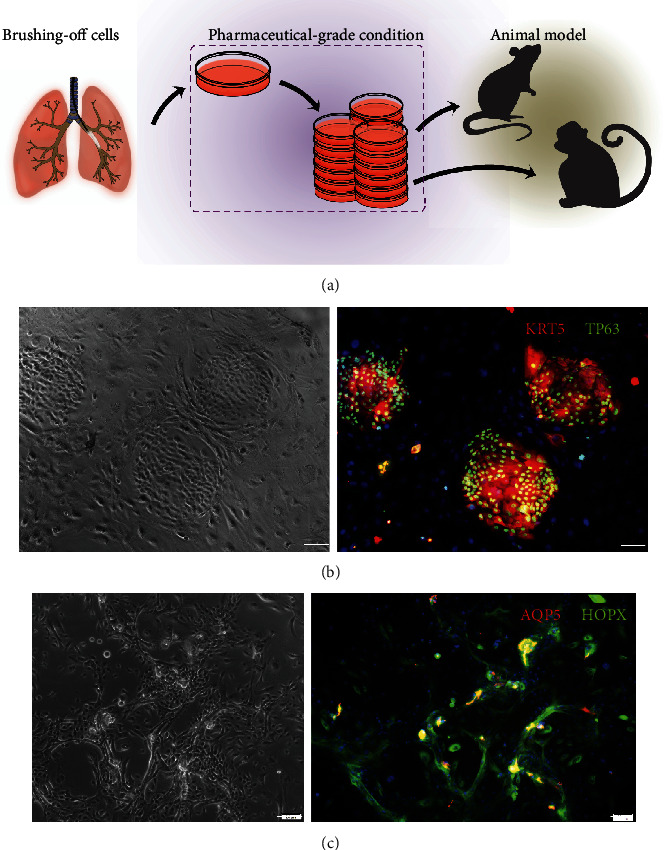
Bronchoscopic isolation of hDASCs from the patient lung. (a) Schematic illustrating the process of clonogenic hDASC isolation and expansion. (b) Clonogenic cells immunostained with hDASC markers KRT5 and P63. (b1) Bright-field imaging. (b2) Immunofluorescence imaging. Scale bar, 100 *μ*m. (c) Differentiation culture of hDASCs in the monolayer on day 7. (c1) Bright-field imaging. (c2) Immunofluorescence imaging. Scale bar, 50 *μ*m.

The distal airway stem/progenitor cells (DASCs), expressing Krt5^+^/P63^+^, have been shown to have a potent regenerative capacity [16, 20, 25]. Here, we isolated human P63^+^/KRT5^+^ DASCs (hDASCs) from the 6th-order airway of lung disease patients by bronchoscopic brushing and then expanded in a culture system as previously described [16] (Figure 1(a)). Briefly, the brush-off sample was dissociated and digested into single-cell suspension that is successfully cloned and propagated under the irradiated 3T3 feeder system with the expression of KRT5^+^ and P63^+^ (Figure 1(b)).

**Figure 2:**
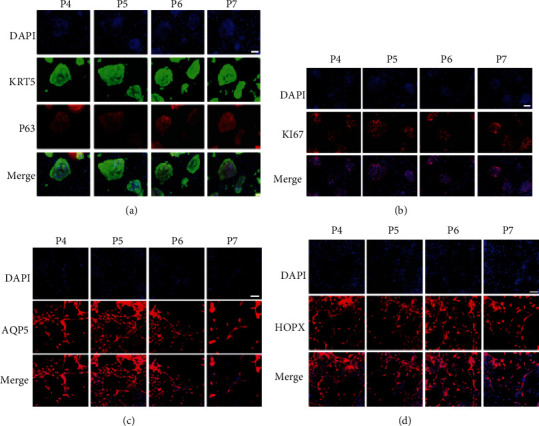
Expansion of hDASCs under the pharmaceutical-grade condition. (a) Clonogenic cells immunostained with hDASC markers KRT5 and P63 among P4 to P7. Scale bar, 100 *μ*m. (b) Clonogenic cells immunostained with proliferative markers KI67 among P4 to P7. Scale bar, 100 *μ*m. (c, d) Immunostaining of indicated AEC1 markers AQP5 and HOPX on monolayer-differentiated DASCs. Scale bar, 100 *μ*m.

Furthermore, to explore the differentiation potency of hDASCs *in vitro*, they were transferred into a feeder-free monolayer differentiation system and gave rise to a few alveolus-like structures lined by thin, highly elongated cells exhibiting AQP5 and HOPX expression, consistent with their type I alveolar epithelial cell (AEC1) identity (Figure 1(c)). To evaluate their clinical potential, isolation, expansion, and quality control of hDASCs were performed under the pharmaceutical-grade condition with all the related components in the culture medium replaced with GMP-grade ones. We have serially monitored the expression of KRT5 and KI67 from passage 4 (P4) to passage 7 (P7) *in vitro*. Among diverse passages, the gross morphology of hDASCs appeared similar and the capacity to express critical identity markers was generally maintained (Figures 2(a) and 2(b)). Consistent with this, amplified human DASCs preserved differentiation potency among serial passage (Figures 2(c) and 2(d)). The above data indicated that the current pharmaceutical-grade DASC expansion system is able to produce an autologous cell population from the lungs under pathophysiological conditions, which maintains robust differentiation capacity.

### 3.2. Safety Evaluation of hDASC Transplantation in Mice

**Figure 3:**
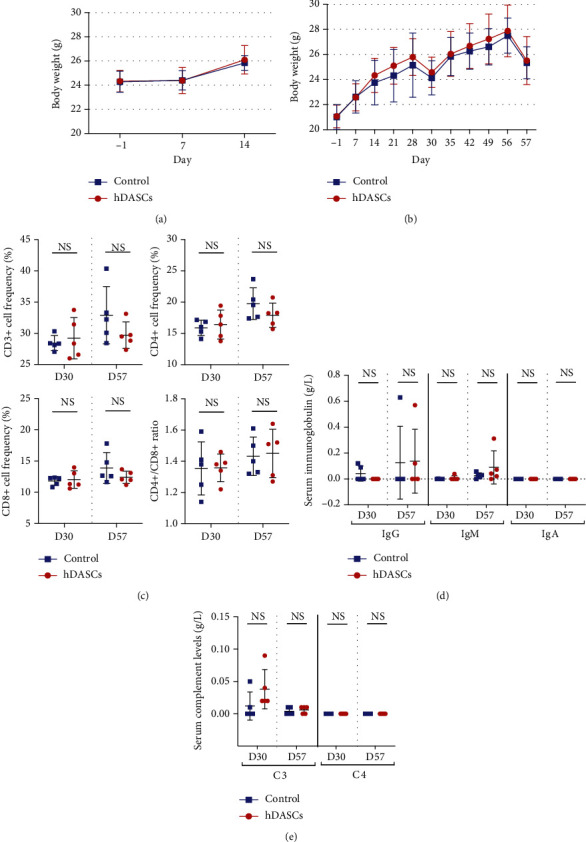
Safety evaluation of hDASC transplantation in mice. (a) Changes in mouse body weight at indicated days in short-term safety assay. *n* = 10 in each group. (b) Changes in body weight at indicated days in long-term safety assay. *n* = 20 in each group. (c) Quantification of lymphocyte subsets (CD3+, CD4+, and CD8+) and CD4+/CD8+ ratio on day 30 and day 57 in long-term safety assay. *n* = 5 in each group. (d, e) Quantification of serum immunoglobulin and serum complement levels on day 30 and day 57 in long-term safety assay. For each test, *n* = 5 in each group.

Our contracted third-party collaborators performed short-term and long-term safety preclinical studies of DASCs according to Good Laboratory Practice (GLP) regulations. A 14-day short-term safety assay showed no mortality or morbidity in NOD-SCID mice after single intratracheal administration of human DASCs at various dose levels (6 × 10^6^–5 × 10^8^ cells/kg). There were no obvious abnormalities in the physical, physiological, or neurological activities of mice. Bodyweight and food/water intake demonstrated weekly fluctuations within the range of control animals (Figure 3(a)).

For the long-term safety study, during the entire 57-day observation period, no mortality was seen in the cell treatment group. Daily recording in physical, physiological, or neurological characteristics demonstrated no appreciable changes in treated animals. Bodyweight demonstrated no significant differences compared to control animals (Figure 3(b)). Quantification of lymphocyte subpopulation, serum immunoglobulins, and complement demonstrated comparable levels in control and experimental groups (Figures 3(c)–3(e)).

### 3.3. Evaluation of the Tumorigenic Potential of hDASC

**Figure 4:**
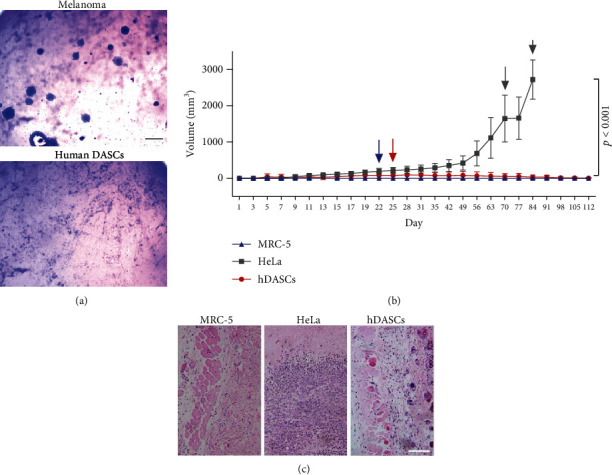
Evaluation of the tumorigenic potential of hDASCs. (a) Soft agar assay of melanoma and hDASCs. Scale bar, 200 *μ*m. (b) Growth curves of subcutaneous tumors in NOD-SCID mice formed by inoculation with either 10^7^ human embryonic lung fibroblast cell MRC-5, 10^6^ HeLa cells, or 10^7^ hDASCs. The blue arrow indicates that 2 mice of the MRC-5 group were euthanized for gross observation and histopathological examination. The red arrow indicates that 2 mice of the hDASC group were euthanized for gross observation and histopathological examination. Grey arrows indicate that 4 mice and 6 mice of the Hela group were euthanized on day 70 and day 84, respectively, because the longest tumor diameter exceeded 20 mm. *n* = 5 in each group. (c) Representative H&E staining of different subcutaneous grafts. Scale bar, 100 *μ*m.

To evaluate that whether the hDASCs were tumorigenic *in vitro*, we assessed the anchorage-independent growth potential of these cells. The data showed that human DASCs were unable to grow in soft agar medium, in contrast that mouse melanoma cells (B16) exhibited robust colony-forming efficiency under identical conditions (Figure 4(a)).

To confirm the tumorigenicity of human DASCs *in vivo*, cells were subcutaneously injected into NOD-SCID mice. The normal human fetal lung fibroblast cell line MRC-5 and cervical cancer cell line HeLa were also injected as the negative and positive controls, respectively. During the 16 weeks of the observation period, all mice implanted with HeLa cells that developed tumors at the injection site, including 4 mice and 6 mice in this group, were euthanized on day 70 and day 84, respectively, due to the chest compression from their large tumors. 8 mice (80%) in the hDASC-implanted group developed subcutaneous nodules with a spontaneous regression by the end of the observation period (Figure 4(b)). Mice with the nodules were sacrificed and histopathological examination confirmed that they were cyst lesions, but not tumors (Figure 4(c)). Altogether the above data indicated that human DASCs produced in the pharmaceutical-grade condition facilities under current protocol are nontumorigenic and safe to be tested in human patients.

### 3.4. Alveolar Regeneration by Intrapulmonary Transplantation of hDASCs

**Figure 5:**
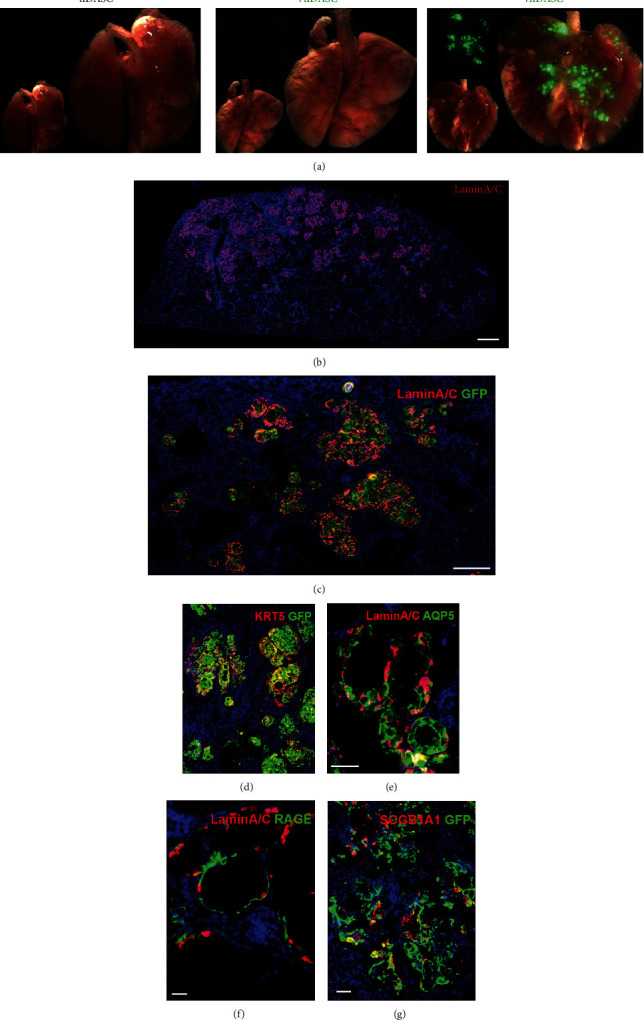
Alveolar regeneration by intrapulmonary transplantation of hDASCs. (a) Bright field and direct fluorescent images of the chimeric lung after GFP+ hDASC transplantation. (a1) Mock-transplanted. (a2) Uninjured lung. (a3) GFP-labeling hDASCs transplanted. (b) Immunofluorescence images of the chimeric lung of anti-Lamin A/C (red) with DNA counterstain (DAPI, blue). Scale bar, 1000 *μ*m. (c, d) Immunofluorescence images of the chimeric lung with GFP and human-specific Lamin A/C and hDASC marker KRT5 Abs. Scale bar, 200 *μ*m. (e, f) Immunofluorescence images of the chimeric lung with human-specific Lamin A/C and AEC1 marker AQP5 and RAGE. Scale bar, 30 *μ*m. (g) Immunofluorescence images of the chimeric lung with human-specific Lamin A/C and SCGB1A1. Scale bar, 50 *μ*m.

To determine whether cloned hDASCs could contribute to lung tissue regeneration *in vivo*, we labeled the cultured cells by GFP-expressing lentivirus and transplanted them into immune-deficient NOD-SCID mouse lungs. Bleomycin was intratracheally instilled to mouse lungs prior to the transplantation. 21 days after the cell transplantation, large-scale GFP+ cell incorporation was observed in parenchymal areas of mouse lungs (Figure 5(a)). Meanwhile, lungs without bleomycin injury showed no incorporation of GFP+ cells posttransplantation, indicating that damage of the lung parenchyma is one of the prerequisites of exogenous hDASC incorporation (Figure 5(a)). After transplantation, the chimerism was further confirmed by human-specific Lamin A/C Ab costaining with GFP in mouse lungs (Figures 5(b) and 5(c)) [26]. The identity of KRT5 expression was also maintained in some engrafted cells 7 days posttransplantation (Figure 5(d)).

Next, we directly assessed the differentiation status of the hDASCs and their progeny by performing immunofluorescence detection of functional lung epithelial cells. 21 days posttransplantation, mature AEC1 markers AQP5 and RAGE [27, 28] were extensively expressed in engrafted human cells, which occupied bleomycin-denuded areas of existent alveoli and formed structures analogous to air sacs in the mouse lung parenchyma (Figures 5(e) and 5(f)). Since AEC1s are the main functional cells that constitute the interface overlying the vascular endothelium essential for gas exchange, the above observations suggested that intratracheal delivery of exogenous hDASCs held potential as a strategy for lung function restoration. In addition, the engrafted cells could also incorporate into the bronchiolar region, where some of them gave rise to SCGB1A1+ Club cells (Figure 5(g)).

### 3.5. Bronchoscopic Delivery of hDASC into the Macaque Lung

**Figure 6:**
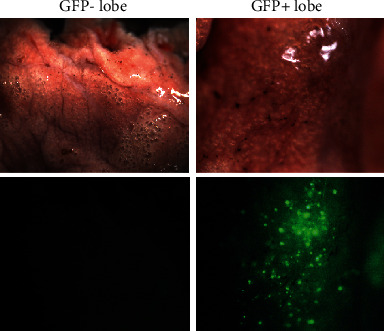
Bronchoscopic delivery of hDASCs into the macaque lung.

To assess the safety and efficacy of the clinical cell delivery, human DASCs were transplanted into a nonhuman primate, cynomolgus macaque (*Macaca fascicularis*) pretreated with a single low dose of total body irradiation (3.2 Gy), causing immune suppression, such as neutropenia and lymphopenia. GFP-labelled DASCs, a total of 40 million cells suspended in PBS, were orthotopically infused into the macaque lung through bronchoscopy following clinical protocols. There was no obvious variety in weight and food-intake during the whole treatment. Also, vital signs and clinical observation of macaque all appeared normal. Seven days posttransplantation, macaque was euthanatized and all organs of it were harvested and the distribution of the GFP signal was monitored. The positive GFP+ cell signal scattered distributed in the transplanted lung lobe area was observed. No discernible GFP signal was detected under a fluorescence stereomicroscope in other organs/tissues such as the liver and kidney (Figure 6 and Table 1). DASC transplantation did not contribute to tumor development, aberrant cell growth, or other related adverse events, suggesting that DASC transplantation procedure might be safe in primates under the quantitative limitation of the rare subject.

## 4. Discussion

The lung, as one of the few organs exposed to the outside, is vulnerable to attack by pathogens, consequently triggering the progressive and inexorable destruction of oxygen exchange surfaces and airway, which is a major threat to human health. Recently, the outbreak of COVID-19, caused by the SARS-CoV-2 virus, has emerged, resulting in death primarily via respiratory failure [29–31]. It is hard to reconstruct the gas exchange surface and respiratory function by the means of current mitigating treatments. Given the scarcity of donor organs, as well as severe sides resulted from immune rejection, the application of lung transplant surgery is constrained [32]. Our previous studies demonstrated lung regeneration in mice following H1N1 influenza virus infection and bleomycin injury, involves distal airway stem cells expressing Trp63 (P63) and Keratin 5, called P63^+^/KRT5^+^ DASCs, to this process. Besides this, other adult lung-specific stem/progenitor cell lineages were also reported to hold great potential as cell therapy candidates, including SCGB1A1+SFTPC+ bronchioalveolar stem cells in the bronchoalveolar duct and SCGB1A1+ Club cells in trachea and upper airways and AEC2 in the alveolar bed [10, 12–15]. Compared with the relatively mature regeneration field, therapeutic potential of lung stem/progenitor cells will be more noteworthy in the future. Here, we only test the human DASCs as a targeted subject and highlight the remarkable generation stability and regenerative capacity of the cloned DASCs *in vitro*.

Stem cell therapy is an emerging therapeutic strategy, as an alternative therapeutical method of organ transplantation [33, 34]. Generally, the autologous cell is the best choice in transplantation, avoiding being attacked by immunocytes. An enormous number of human DASCs are needed in clinical transplantation, considering rare numbers of hDASCs *in vivo*; their extensive *in vitro* expansion is required. Whereas, such expansion raises simultaneously some risks such as genetic and epigenetic changes. Cell proliferation *in vitro* could result in the occurrence of mutations and chromosomal aberrations, eventually leading to tumorigenicity. Thus, the safety and stability evaluation of expansion was elaborated *in vitro* and *in vivo*. Clones under the pharmaceutical-grade culture maintained their self-renewal, potency properties, and uniform identification *in vitro* amongst passages, which is crucial for cell quality control in further clinical application.

Immune-deficient mouse NOD-SCID and irradiated nonhuman primate cynomolgus macaque were the receptors of human DASCs to ameliorate the immunological rejection from disparate species. The chimeric of human-mouse lung indicates that hDASC pedigrees contributed multiple epithelial types, including AEC1 as well as bronchiolar secretory cells, to the regenerating distal lung, which is a general species-crossing repair manner following large-scale, acute lung damage. Moreover, an unconventional route of cell administration utilizing a portable fiberoptic bronchoscope in macaque was applied in the current study. Engraftment of hDASCs into macaque did not cause anaphylaxis or tumorigenic. Admittedly, only a relatively small number of macaque subject was used for the sake of animal welfare. Whether human DASCs could generate to the chimeric of the human-macaque lung would be the problem that we will address.

In summary, our data highlighted the hDASCs maintaining stem cell/progenitor properties during expansion *in vitro* under the pharmaceutical-grade condition as a candidate of cellular therapy. The chimeric of the human-mouse lung indicated hDASC pedigrees contributed multiple epithelial types, including AEC1 as well as bronchiolar secretory cells, to the regenerating distal lung.

## Figures and Tables

**Table 1:** Fluorescent examination of macaque organs.

Organs	GFP signal
Adrenal glands	NA
Aorta	NA
Bladder	NA
Bone and marrow (femur and sternum)	NA
Brain	NA
Epididymis	NA
Esophagus	NA
Eye and optic nerve	NA
Heart	NA
Kidney	NA
Lacrimal gland	NA
Large intestine (cecum, colon, and rectum)	NA
Larynx	NA
Liver	NA
Lung and bronchi	+
Mammary glands	NA
Pancreas	NA
Peyer patch	NA
Pituitary gland	NA
Prostate gland	NA
Salivary glands	NA
Sciatic nerve	NA
Skeletal muscles (biceps femoris)	NA
Skin (perimammary glands)	NA
Small intestine (duodenum, jejunum, and ileum)	NA
Spermathecal glands	NA
Spinal cord (cervical, thoracic, lumbar)	NA
Spleen	NA
Stomach (pancreatic stomach and nonpancreatic stomach)	NA
Submandibular and mesenteric lymph nodes	NA
Testes	NA
Thymus	NA
Thyroid and parathyroid glands	NA
Tongue	NA
Trachea	NA

## Data Availability

The data used to support the findings of this study are available from the corresponding author upon request.
